# Ulcer in a dialysis patient: Calciphylaxis or something simpler

**DOI:** 10.1002/ccr3.5318

**Published:** 2022-09-12

**Authors:** Amr Mohamed, Salem Thabet

**Affiliations:** ^1^ 6932 Department of Internal Medicine Rochester General Hospital Rochester New York USA

**Keywords:** dialysis, pruritis, ulcer, uremia

## Abstract

Ulcers in dialysis patients have multiple etiologies; uremic pruritis is common in dialysis patients and is associated with poor outcomes; however, it is more likely to be underdiagnosed as we usually think about more serious etiologies as calciphylaxis. Here, we present a case where uremic pruritis was the leading diagnosis.

## Graphical abstract

In patients with end‐stage renal disease presenting with lower extremity ulcers, the differential diagnoses include vasculitis, infectious process, and calciphylaxis. Nevertheless, simple causes, such as uremic pruritus,should also be remembered.

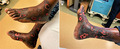

We present a 56‐year‐old man with a past medical history of end‐stage renal disease on regular hemodialysis, streptococcal endocarditis 4 years ago, COVID‐19 pneumonia 3 months ago presented to the ED with a complaint of wounds in both legs, as shown in Figure 1.

**FIGURE 1:**
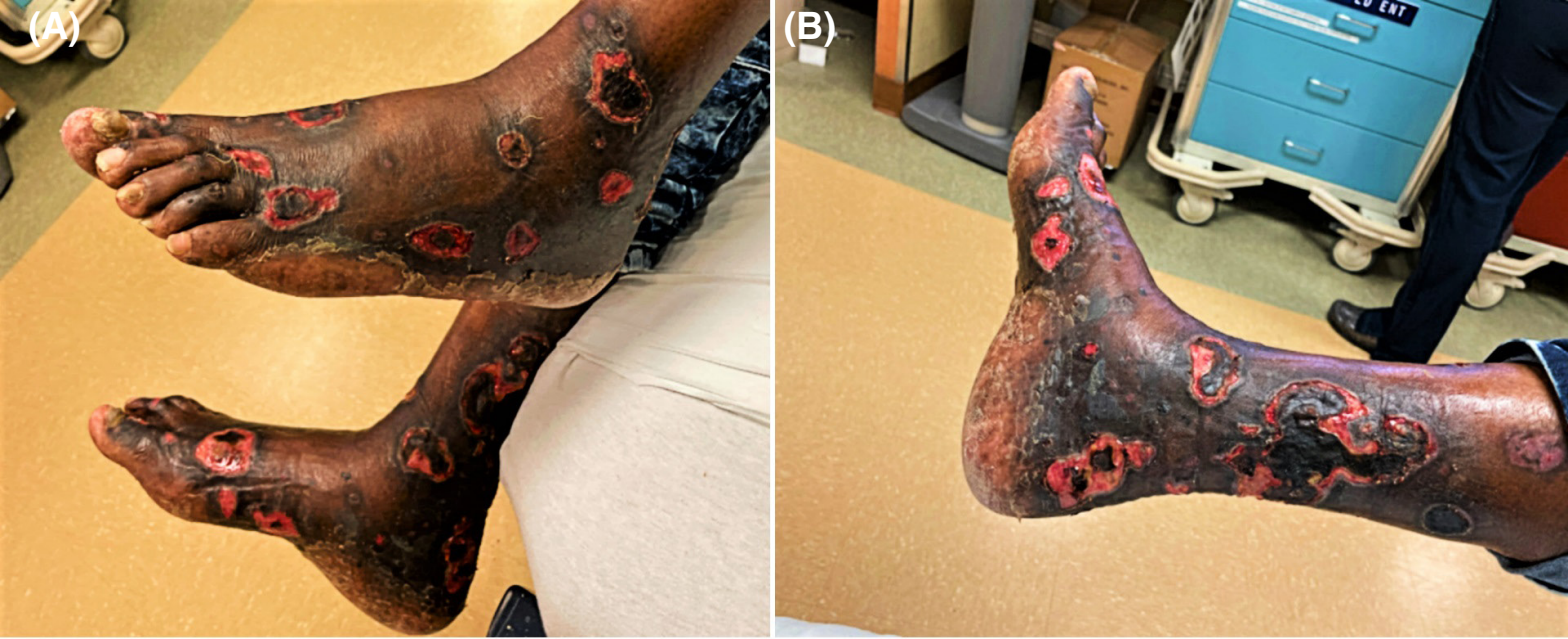
Bilateral lower extremity ulcers in geographic distribution on both lower extremities

He reported severe itching for 2 months, and the ulcers started 3 weeks before presentation. The ulcers were initially painful, later painless, initially on the right foot, later on, both right and left foot, and the lower legs on both sides. He denied IV drug abuse, and his drug screen had been negative. His physical examination had been normal other than the leg ulcers, with no systemic signs of infection and no fevers.

Infective endocarditis had been ruled out with negative blood cultures and a negative transthoracic echocardiogram. A full autoimmune workup was negative.

Given that the clinical picture was unclear, multiple skin biopsies were performed, showing no evidence of vasculitis, infectious process, or calciphylaxis. The epidermis adjacent to the ulcer is reactive/hyperplastic, and the changes were suggested to be secondary to CKD‐associated pruritus. The patient received Triamcinolone cream with antihistaminic and local wound care, and the ulcers improved, as shown in Figure 2 in the supplementary section.

**FIGURE 2:**
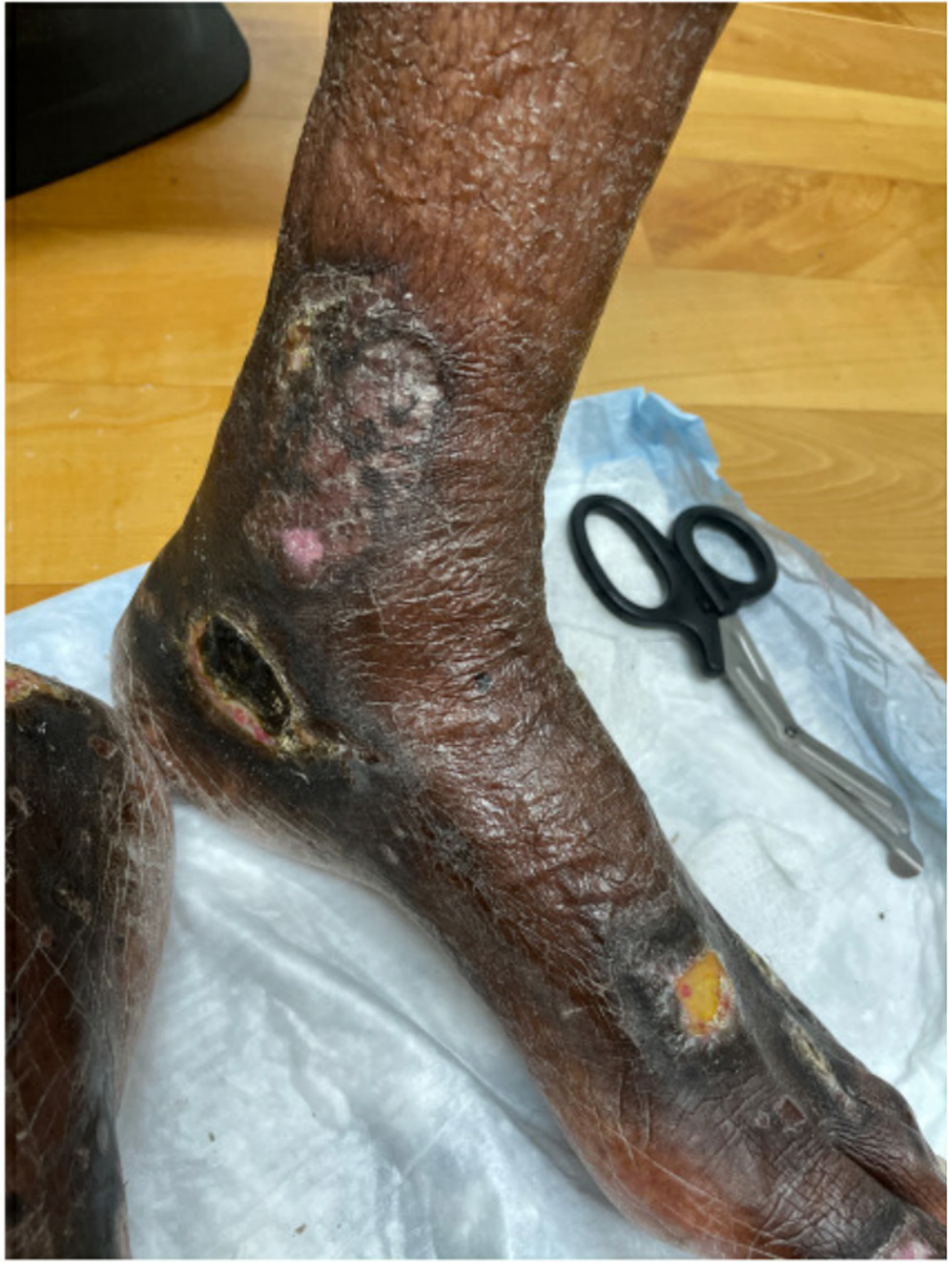
Marked improvement in the lower extremity ulceration after adequate treatment

The key clinical message is that in patients with end‐stage renal disease presenting with lower extremity ulcers, the differential diagnoses include vasculitis, infectious process, and calciphylaxis. Nevertheless, simple causes, such as uremic pruritus,^1^ should also be remembered.

## CONFLICT OF INTEREST

None.

## AUTHOR CONTRIBUTIONS

Both authors contributed to the data collection and case presentation.

## ETHICAL APPROVAL

Our institution does not require ethical approval for reporting individual cases or case series. The patient received standard of care treatment and was not subject to any experimentation.

## CONSENT

Patient written permission had been obtained to publish his images. The approval had been documented in his electronic medical record.

## Data Availability

The data that support the findings of this study are available from the corresponding author upon reasonable request.
